# Supplementary material for: Quantitative measurement of antibiotic resistance in Mycobacterium tuberculosis reveals genetic determinants of resistance and susceptibility in a target gene approach
Source: Res Sq. 2023 Oct 2:rs.3.rs-3378915. Preprint. [Version 1] doi: 10.21203/rs.3.rs-3378915/v1 (PMC10602118; doi:10.21203/rs.3.rs-3378915/v1)
Supplement: Supplement 1 [file NIHPPrs3378915v1-supplement-1.pdf]

Supplementary Files

This is a list of supplementary files associated with this preprint. Click to download.

- MembersoftheCRyPTICconsortium.docx
- SuppTables230305.xlsx
- NCOMMS2306781Ars.pdf
- FigS1.png
- FigureS2embed.ai

- FigS3.png
- FigS42.png
- FigS5.png
